# Plasma lipids, amino acids, and their metabolic pathways as potential biomarkers for differential diagnosis of cold and heat syndrome asthma in children: a preliminary study

**DOI:** 10.3389/fped.2025.1549431

**Published:** 2025-07-23

**Authors:** Yi Sun, Lianzhan Huang, Wenhui Yao, Zhengguang Chen, Dong Cui, Xuansheng Ding, Lisheng Wan

**Affiliations:** ^1^ Department of Traditional Chinese Medicine, Shenzhen Children's Hospital, Shenzhen, China; ^2^ Department of Pediatrics, Beijing Anzhen Nanchong Hospital of Capital Medical University & Nanchong Central Hospital, Nanchong, Sichuan, China; ^3^ School of Basic Medicine and Clinical Pharmacy, China Pharmaceutical University, Nanjing, China

**Keywords:** children asthma, cold asthma, heat asthma, TCM syndromes, metabolomics, plasma biomarkers

## Abstract

**Background:**

Childhood asthma has a significant effect on growth and development. Traditional Chinese Medicine (TCM) has notable advantages in asthma treatment; however, a modern scientific basis for the differentiation of cold and heat syndromes in asthma remains lacking.

**Methods:**

This study employed non-targeted metabolomics to analyze the plasma metabolic profiles in children aged 5–14 years with cold or heat syndrome asthma. Plasma metabolites were examined to identify and compare metabolic differences among children with asthma and healthy controls, as well as between cold and heat asthma syndromes, with the aim of uncovering potential biomarkers and providing a foundation for differential diagnosis.

**Results:**

Of the 92 participants, 48 had cold syndrome asthma, 14 had heat syndrome asthma, and 30 were healthy controls. A total of 50 differential plasma metabolites were identified between the TCM asthma syndrome groups and healthy controls in both positive and negative ion modes. These metabolites were primarily phospholipids and amino acids enriched in the lipid metabolism, amino acid metabolism, and glucose metabolism pathways. Furthermore, 18 differential metabolites were identified between the cold and heat asthma groups, with significant enrichment in the amino acid metabolic pathways. Notably, 36 common differential metabolites that mainly were lipids, amino acids and its related metabolites between cold asthma and heat asthma, cold asthma and the healthy group, and heat asthma and the healthy group were identified of which can be considered as biomarkers.

**Conclusions:**

Lipids, amino acids, and their associated metabolic pathways have been identified as potential biomarkers for distinguishing cold and heat asthma syndromes in children. These findings contribute to the modern interpretation of TCM syndrome differentiation and may support the evaluation of the therapeutic effects of TCM-based asthma treatment.

## 1 Introduction

Bronchial asthma, commonly referred to as asthma, is the most prevalent chronic respiratory disease in children. Data show that its incidence is increasing annually worldwide (1, 2). However, owing to its recurrent nature, it seriously affects the physical and mental health of children and imposes a heavy economic burden on both families and society (3). Moreover, the pathogenesis of asthma is diverse and complex, influenced by climate change, environmental pollution, and genetic heterogeneity (4, 5), and involves chronic airway inflammation (6), immune responses, airway hyperresponsiveness, and genetic factors (7). Currently, the management and control of asthma remain inadequate, particularly in low-resource settings (2). Therefore, this issue warrants extensive research.

In Traditional Chinese Medicine (TCM) theory, asthma is classified under the category of “*Xiao* disease.” According to TCM guidelines and expert consensus, pediatric asthma is a recurrent lung disease characterized by wheezing, coughing, and rales in the throat. Clinical episodes present symptoms, such as throat rales, shortness of breath, coughing, chest tightness, and prolonged expiration. In severe cases, patients may be unable to lie down and may experience dyspnea, open-mouth breathing, shoulder lifting, body shaking, lip cyanosis, and irritability. Symptoms often occur or worsen in the early morning or at night (8–10). This disease corresponds to bronchial asthma in children in Western medicine (10). Asthma can be divided into three distinct phases: exacerbation, relocation, and remission. The exacerbation phase is characterized by a sudden onset of symptoms, such as wheezing, coughing, shortness of breath, chest tightness, or the rapid worsening of existing symptoms. This phase corresponds to the acute attack stage in Western medicine, and includes cold asthma syndrome, heat asthma syndrome, and external cold with internal heat syndrome (8). Cold and heat asthma are the most common clinical phenotypes.

The TCM differentially diagnostic criteria for cold asthma and heat asthma can be seen in Table 1 (8, 11). Compared to adults, TCM holds that children have immature organs and insufficient *Qi*, making them more vulnerable to external pathogens. The Plain Questions—Theory of Wind states, “Wind is the root of all diseases.” In TCM theory, “wind evil” is a type of pathogenic *xie Qi*, characterized by sudden onset, rapid changes, and migration of symptoms—resembling natural wind. When wind evil invades the human body, it can affect organs such as the lungs, spleen, and kidneys, leading to various diseases. Wind-related syndromes can be classified as external (originating from nature) or internal (resulting from visceral dysfunctions). In children, the lungs regulate fluid distribution, the spleen governs transportation and transformation, and the kidneys lack the ability to efficiently vaporize fluids. These deficiencies contribute to phlegm accumulation and the formation of asthma roots. Clinically, TCM categorizes asthma into cold and heat syndromes based on these manifestations (12). TCM has been widely used to treat asthma (13). Despite its notable efficacy and safety, a universally accepted TCM diagnostic standard is currently lacking. Therefore, a comprehensive and scientific explanation of the nature and connotations of TCM syndromes is essential.

**Table 1:** TCM syndrome differentiation of cold and heat asthma in children.

TCM syndrome	Differential diagnosis	Definition description
Cold asthma	Symptoms	Wheezing in the throat is like the sound of a water cock, shortness of breath, wheezing and suffocating, chest tightness is like stuffiness, sputum color is white and foamy, mouth is not thirsty or thirsty for hot drinks, cold limbs are afraid of cold, face is blue and gloomy, easy to recurrent in cold weather or cold.
	Tongue pulse	The tongue coating is white and smooth, the body is fat, and the pulse string is tight or floating.
	TCM pathogenesis	Deficiency of physical body, cold phlegm crouching in the lung, triggered by sensation, phlegm and *Qi* blocking, disruption of lung's propagation and descent.
Heat asthma	Symptoms	The sputum in the throat is as loud as roar, both wheezing and shortness of breath, cough and choking, expectoration is yellow or white, thick, muddy and viscous, and cough and vomiting is unfavorable, accompanied by hard mouth or not, red sweat on the face, or body heat.
	Tongue pulse	The tongue is red, the coating is yellow and greasy, and the pulse is slippery or string slippery
	TCM pathogenesis	Constitution with insufficiency, phlegm-heat obstructing lung, airway obstruction, impaired purgative descending of lung *Qi*.

Metabolomics is the study of the types, quantities, and variations in endogenous metabolites in organisms following stimulation or disturbance (14). Metabolites are the end products of gene and protein activities and can promptly, sensitively, and accurately reflect the functional state of an organism in response to external stimuli. As such, they are considered “biological phenotypes” of physiological functions (15). In recent years, increasing evidences have shown that metabolomics can be applied to study the pathogenesis, diagnosis, and treatment of asthma and other diseases (16–20). Some studies have applied metabolomics to explore the TCM syndromes of asthma (12), indicating significant metabolic disturbances in patients with asthma. However, few studies have specifically focused on TCM syndromes in pediatric asthma. Therefore, investigating potential biomarkers associated with TCM asthma syndromes is of great significance for clinical diagnosis.

In this study, we aimed to identify plasma biomarkers in children with asthma during acute exacerbation and screen for differential metabolites that may help elucidate the pathogenesis of asthma. We recruited children diagnosed with cold and heat asthma syndromes based on the TCM criteria from Shenzhen Children's Hospital, along with healthy children as a control group. Plasma samples were collected and analyzed using liquid chromatography–mass spectrometry (LC-MS)-based metabolomics.

## 2 Materials and methods

### 2.1 Study population and study design

A total of 62 patients aged 5–14 years with asthma were recruited from Shenzhen Children's Hospital between January 2016 and September 2017. Asthma subtypes were classified based on the diagnostic criteria for pediatric bronchial asthma and TCM syndrome classification for pediatric asthma. The final diagnosis was made through unanimous agreement between the two chief physicians.

The exclusion criteria were as follows: (1) patients whose diagnosis did not meet the inclusion criteria; (2) those with confirmed congenital or acquired metabolic disorders; (3) patients with comorbid conditions, such as toxic encephalopathy, respiratory failure, or heart failure; (4) individuals with diagnosed mental illness; (5) patients with severe primary diseases affecting the heart, liver, kidneys, or hematopoietic system; and (6) patients undergoing treatment with glucocorticoids, immunosuppressants, or participating in clinical drug trials.

Thirty age- and sex-matched children without any evidence of asthma were recruited as healthy controls. This study was approved by the Ethics Committee of Shenzhen Children's Hospital. Written informed consent was obtained from all participants and their legal guardians.

### 2.2 Sample collection and processes

Two milliliters of morning fasting venous blood were collected from each participant using a disposable heparin anticoagulant tube. The samples were centrifuged at 3,000 rpm for 10 min, and the resulting plasma was transferred to cryopreservation tubes and stored at −80 °C to avoid repeated freeze–thaw cycles.

Prior to the analysis, the plasma samples were thawed at room temperature. Then, 40 μl of plasma was transferred to a new tube and 120 μl of cold precipitant (dichloromethane/methanol, 3:1, v/v) was added. The mixture was vortexed for 1 min, left to stand at room temperature for 10 min, and stored at −20 °C overnight. The next day, the samples were centrifuged at 4,000 rpm for 30 min at 4 °C. From each sample, 25 μl of the supernatant was transferred to a new centrifuge tube and 225 μl of a lipid complex diluent (isopropanol:acetonitrile:water, 2:1:1, v/v/v) was added.

For quality control (QC), 20 μl of each sample was pooled to obtain a QC sample. Before initiating the analysis, ten QC samples were run to equilibrate the instrument. Subsequently, one QC sample was injected for every 10 test samples, and three QC samples were run at the end of the sequence to monitor the system stability.

### 2.3 Untargeted metabolomics analysis

Untargeted metabolomic analysis was performed using liquid chromatography coupled with mass spectrometry. Chromatographic separation was carried out on an ACQUITY Ultra-Performance Liquid Chromatography (UPLC) system (2777C, Waters, UK). The samples were analyzed in the order determined by the instrument sequence. A reversed-phase ACQUITY UPLC HSS T3 column (100 mm × 2.1 mm, 1.8 μm, Waters, UK) was used. The column oven temperature was maintained at 40 °C. The mobile phases consisted of solvent A (water with 0.1% formic acid) and solvent B (acetonitrile with 0.1% formic acid) at a flow rate of 0.4 ml/min. Gradient elution was programmed as follows: 0–1 min, 99% A; 1–3 min, 1%–15% B; 3–6 min, 15%–50% B; 6–9 min, 50%–95% B; 9–10 min, 95% B; and 10.1–12 min, 99% A. The injection volume for each sample was 10 μl.

Mass spectrometry analysis was conducted using a Xevo G2-XS QTOF system (Waters, UK) operated in both the positive and negative ion modes. In positive ion mode, the capillary voltage and sample cone voltage were set to 0.25 kV and 40 V, respectively. In the negative-ion mode, the voltage was set to 2 kV and 40 V. Data were acquired in the centroid MSE mode. The TOF mass range was set to 50–1,200 Da with a scan time of 0.2 s. For MS/MS detection, all precursor ions were fragmented at 20–40 eV, with a scan time of 0.2 s. During the acquisition, a lock mass (LE) signal was collected every 3 s to ensure mass accuracy. In addition, one QC sample was analyzed every 10 experimental samples to monitor the stability and reproducibility of the LC-MS system.

### 2.4 Data collection and metabolite identification

Untargeted raw data were processed and identified using the commercial software, Progenesis QI (version 2.2). Local polynomial regression fitting for signal correction based on quality control (QC) sample data, referred to as QC-based robust LOESS signal correction (QC-RSC), was used to adjust the real sample signals. This method is recognized as an effective approach for signal correction in metabolomic data analyses (21). An internal standard, in combination with the QC-RSC approach, was used to calibrate the peak areas of the plasma samples. After normalization, the data acquired from both the positive and negative ion modes were merged into a single dataset for downstream metabolite analysis.

Multivariate statistical analyses, including principal component analysis (PCA) and partial least-squares discriminant analysis (PLS-DA), were performed using MetaboAnalyst or metaX software (22). The key performance metrics for evaluating the PLS-DA model included *R*^2^*Y* and *Q*^2^ values. *R*^2^*Y* indicates the proportion of variation in the dependent variable *Y* explained by the model, whereas *Q*^2^ reflects the predictive ability of the model. In general, *R*^2^*Y* and *Q*^2^ values closer to 1 suggest a more robust model, with *Q*^2^ > 0.5 indicating good predictive performance.

Variable Importance in Projection (VIP) scores were extracted from the PLS-DA model to evaluate the contribution of individual metabolites to the group discrimination. These VIP values were used to identify differentially expressed metabolites.

Univariate statistical analyses, including *t*-tests and fold-change analysis, were conducted to further evaluate metabolite differences. The resulting *p*-values were adjusted using the false discovery rate (FDR) method to obtain q-values. Differential ions were identified based on the combined criteria of VIP ≥1, fold-change ≥1.2 or ≤0.83, and *q*-value <0.05.

Differential metabolites identification were firstly identified via Progenesis QI (version2.2) software, and further validated using the Human Metabolome Database (HMDB, https://hmdb.ca/) (23). Metabolite candidates were refined based on MSE fragmentation data, and non-endogenous metabolites were excluded. Finally, a *t*-test was performed on the relative abundance of endogenous metabolites, and those with *P* < 0.05 were considered significantly different.

### 2.5 Pathway analysis

Pathway enrichment analysis was performed using the MetaboAnalyst database (https://www.metaboanalyst.ca/, version 4.0) (24). Metabolic pathway analysis was conducted to identify specific metabolites and explore the potential pathogenesis of bronchial asthma as well as the metabolic differences between the cold asthma and heat asthma groups.

## 3 Results

### 3.1 Participant characteristics

A total of 92 participants were included, comprising 62 children with asthma and 30 healthy controls. Among children with asthma, 48 were diagnosed with cold syndrome asthma (cold asthma group) and 14 with heat syndrome asthma (heat asthma group). Healthy children served as the controls.

There were no significant differences between the groups in terms of age, sex, or clinical examination results. Additionally, no significant differences were observed in the duration of acute asthma exacerbation, lung function, or family history of allergic diseases (Table 2).

**Table 2:** Clinical characteristics of the study population.

Clinical characteristics	Healthy control	Patients	
		Cold asthma	Heat asthma
Numbers (*n*)	30	48	14
Male (*n*)	25	37	11
Female (*n*)	5	11	3
Age (year)	7.17 ± 1.89	7.47 ± 2.32	6.61 ± 2.14
Course of acute asthma attack (day)	/	4.53 ± 4.50	5.69 + 5.89
Lung function (abnormal/normal/unknown)	/	34/0/14	8/0/6
Family history of allergic diseases (Yes/No)	/	25/23	5/9

Data are presented as mean ± standard deviation (SD) or as counts (*n*). One-way analysis of variance (ANOVA) was used to compare age distributions among the groups, and the chi-square test was used to compare sex distributions.

### 3.2 Plasma profile of non-targeted metabolomics analysis

**Figure 1:**
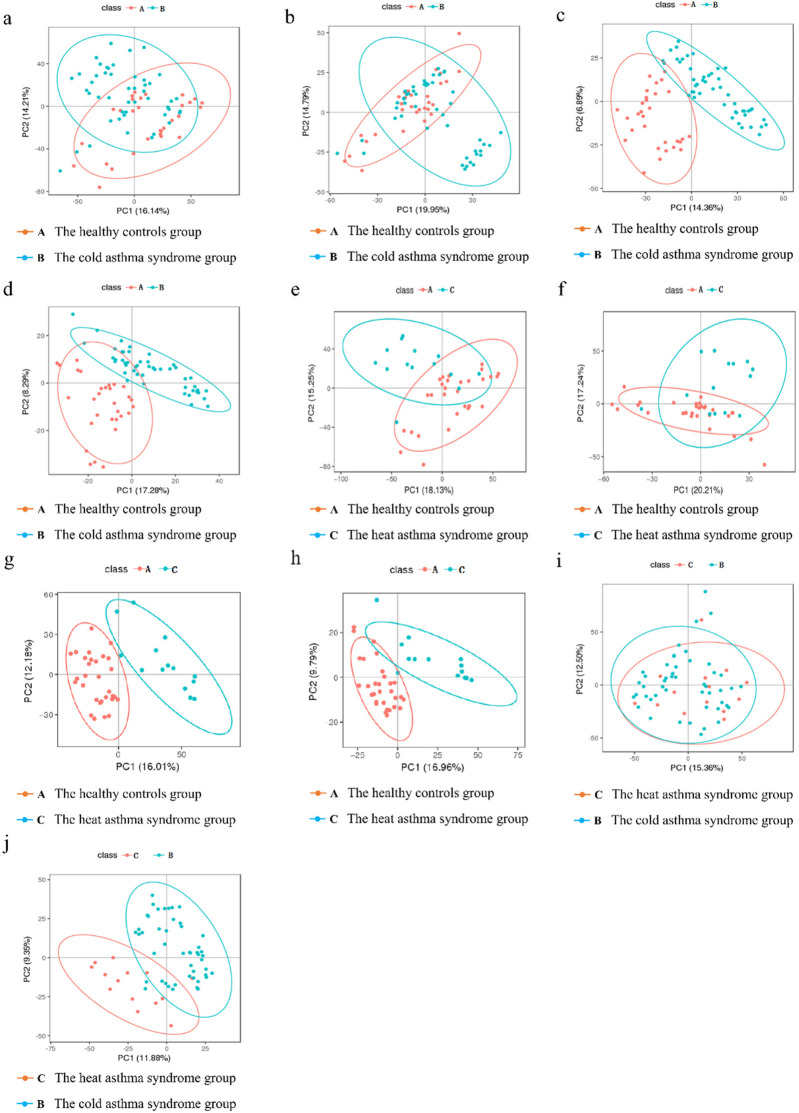
Multivariate analysis of plasma metabolites in different groups. **(a,b)** PCA plots in the positive ion mode **(a)** and negative ion mode **(b)** between the healthy controls group and the cold asthma group. **(c,d)** PLS-DA plots in positive ion mode [**(c)** *R*^2^*Y* = 0.819, *Q*^2^ = 0.683] and negative ion mode [**(d)** *R*^2^*Y* = 0.741, *Q*^2^ = 0.481] between the healthy controls and cold asthma groups. **(e,f)** PCA plots in positive ion mode **(e)** and negative ion mode **(f)** between the healthy controls group and the heat-asthma group. **(g,h)** PLS-DA plots in positive ion mode [**(g)** *R*^2^*Y* = 0.842, *Q*^2^ = 0.707] and negative ion mode [**(h)** *R*^2^*Y* = 0.812, *Q*^2^ = 0.575] between the healthy controls and heat asthma groups. **(i,j)** PCA **(i)** and PLS-DA [**(j)** *R*^2^*Y* = 0.616, *Q*^2^ = 0.627] plots between the cold and heat asthma groups in the positive ion mode. (In the figures, class A represents the healthy controls group; class B represents the cold asthma syndrome group; class C represents the heat asthma syndrome group). In the figure, PC1 and PC2 represent the first and second principal components, respectively. The number in parentheses represents the proportion of the principal component that can integrate the original information. The score graph reflects the distribution of each sample in the coordinate system composed of PC1 and PC2, mainly observing the trend of inter group separation.

Plasma metabolites were analyzed in the healthy control, cold asthma, and heat asthma groups. Principal component analysis in both positive and negative ion modes revealed a discernible separation trend between the healthy control group and the cold asthma group, as well as between the healthy control group and the heat asthma group. However, complete separation was not observed (Figures 1a,b,e,f). In contrast, PLS-DA demonstrated clear separation between the groups, indicating significant differences in plasma metabolite profiles between the healthy control group and the cold asthma group, as well as between the healthy control group and the heat asthma group during asthma exacerbation (Figures 1c,d,g,h).

Additionally, we compared the metabolite profiles of the cold and heat asthma groups. Few differential ions were identified in the negative ion mode; therefore, the subsequent analysis focused on the positive ion mode. PCA revealed no obvious separation trend between the cold and heat asthma groups (Figure 1i), whereas PLS-DA showed a distinct separation (Figure 1j), suggesting substantial metabolic differences between the two asthma syndromes. These findings indicate that plasma metabolites are differentially expressed not only between asthmatic and healthy children, but also between children with cold and heat asthma syndromes.

### 3.3 Identification of the differential metabolites

**Figure 2:**
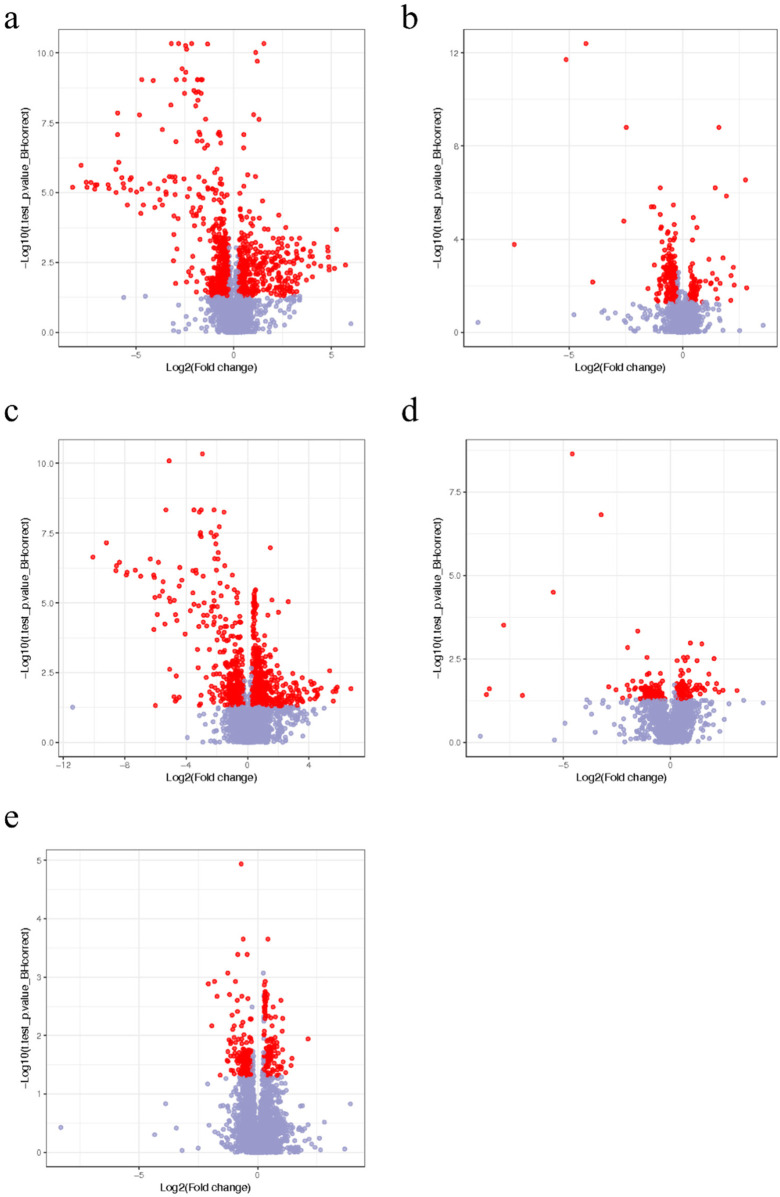
Volcano plots of the identification of differential ions in the plasma samples between the healthy control, cold, and heat asthma groups in the positive and negative ion modes. Volcano plot of the differential ions in the positive ion mode **(a)** and negative ion mode **(b)** between the healthy control and cold asthma groups. Volcano plot of the differential ions in positive mode **(c)** and negative ion mode **(d)** between the healthy control and heat asthma groups. **(e)** Volcano plot of the differential ions in positive ion mode between the cold and heat asthma groups. The red points in the heat plots represent the difference multiples of the differential metabolites that were both ≥1.2 or ≤0.83, and *q*-value <0.05. The blue points represent other metabolites.

Total ion chromatogram (TIC) results under positive and negative ions model of differential metabolites identification and the quality control samples were presented in the Supplementary Figure S1). Differential metabolites between the healthy control group and the cold asthma group were identified using the criteria of fold change ≥1.2 or ≤0.83 and *q*-value <0.05, respectively (Figures 2a,b). A total of 50 differential metabolites were identified: 33 in positive ion mode and 17 in negative ion mode. As shown in Table 3, compared to the healthy control group, plasma levels of trihexosylceramide, diglyceride, triglyceride, and prostaglandin A1 were significantly elevated in the cold asthma group. In contrast, the levels of metabolites such as fructose, vanillic acid, uric acid, fumarate acetoacetate, galactose alcohol, and inositol phosphate were significantly decreased.

**Table 3:** List of differentially expressed metabolites in children with cold asthma and healthy controls.

Mode	Metabolites name	Molecular formula	*m/z*	VIP	Fold change	q-value
ESI+	Fructose	C_12_H_22_O_11_	360.149	2.366	0.54	<0.001
ESI+	2-Hexenyl-6-methoxy-1,4-benzoquinone	C_37_H_54_O_3_	529.4	4.973	0.57	9.72 × 10^−10^
ESI+	2′-deoxycytidine 5′-triphosphate	C_9_H_16_N_3_O_13_P_3_	489.983	1.619	0.59	0.002
ESI+	Vitamin A	C_20_H_30_O	304.263	1.514	0.66	0.004
ESI+	Decanoyl carnitine	C_17_H_33_NO_4_	316.249	2.68	0.68	4.61 × 10^−11^
ESI+	3-hydroxydecanoyl carnitine	C_18_H_35_NO_5_	328.246	2.198	0.67	1.99 × 10^−10^
ESI+	Arginine	C_6_H_14_N_4_O_2_	175.12	1.825	0.67	0.004
ESI+	3-hydroxydodecyl carnitine	C_19_H_37_NO_5_	342.264	1.956	0.72	1.61 × 10^−8^
ESI+	Monoacylglycerol ester	C_21_H_38_O_4_	337.273	1.159	9.61	0.004
ESI+	Corticosterone	C_21_H_34_O_4_	368.28	1.989	0.75	<0.001
ESI+	Prostaglandin A1	C_20_H_32_O_4_	319.228	1.141	9.55	0.008
ESI+	20-hydroxyarachidonic acid	C_20_H_32_O_3_	303.233	1.462	9.5	0.003
ESI+	Trihexose ceramide	C_54_H_99_NO_18_	1,097.726	1.322	10.15	0.016
ESI+	Androstenol	C_19_H_30_O	257.227	1.027	10.81	0.011
ESI+	Pregnanetriol	C_21_H_36_O_3_	337.273	1.159	9.61	0.004
ESI+	Linolenic acid	C_18_H_30_O_2_	301.212	1.297	1.75	<0.001
ESI+	3-Hydroxy-9-Octadecyl Carnitine	C_25_H_47_NO_5_	424.343	1.6	0.75	0.003
ESI+	Vitamin A2	C_20_H_28_O	285.223	1.184	9.51	0.001
ESI+	Lactoside ceramide	C_48_H_91_NO_13_	872.653	1.232	8.07	<0.001
ESI+	Lysophosphatidylethanolamine	C_25_H_48_NO_7_P	544.278	1.149	0.61	0.006
ESI+	Lysophosphatidylcholine	C_22_H_46_NO_7_P	468.309	1.162	0.73	<0.001
ESI+	Cardiolipin	C_73_H_142_O_17_P_2_	1,371.001	1.243	1.5	0.038
ESI+	Triglyceride	C_59_H_96_O_6_	923.717	1.223	10	0.007
ESI+	Diglyceride	C_39_H_66_O_5_	597.488	3.621	10.1	<0.001
ESI+	Cholesteryl ester	C_47_H_78_O_3_	729.561	1.147	9.96	0.003
ESI+	Ceramide	C_48_H_91_NO_13_	872.653	1.232	8.07	<0.001
ESI+	Phosphatidylglycerol	C_46_H_81_O_10_P	842.59	1.533	5.07	0.028
ESI+	Phosphatidylethanolamine	C_43_H_78_NO_8_P	750.541	1.729	5.49	0.002
ESI+	Monoacylglycerol ester	C_21_H_38_O_4_	355.286	4.769	6.3	<0.001
ESI+	Ganglioside GA2	C_56_H_104_N_2_O_18_	1,075.728	5.165	6.31	<0.001
ESI+	Galactosylceramide	C_40_H_77_NO_8_	682.561	1.166	6.66	0.005
ESI+	Sphingomyelin	C_49_H_97_N_2_O_6_P	841.714	1.142	9.04	<0.001
ESI+	Phosphatidylserine	C_42_H_82_NO_10_P	814.564	1.242	4.22	0.007
ESI-	Dihydropterin triphosphate	C_9_H_16_N_5_O_13_P_3_	479.969	2.111	0.58	0.002
ESI-	Biotin	C_10_H_16_N_2_O_3_S	279.06	1.772	0.56	0.013
ESI-	Isodeoxycholic acid	C_24_H_40_O_4_	377.272	1.037	10.18	0.031
ESI-	2-Stearylglycerol phosphate inositol	C_27_H_53_O_12_P	599.319	1.469	1.32	0.014
ESI-	Lysophosphatidylethanolamine	C_23_H_46_NO_7_P	478.293	2.032	0.65	0.018
ESI-	Triglyceride	C_59_H_94_O_6_	883.688	1.287	8.53	0.01
ESI-	Phosphatidylethanolamine	C_53_H_104_NO_8_P	948.722	1.492	9.23	0.008
ESI-	Phosphatidylcholine	C_41_H_80_NO_8_P	744.556	2.087	8.24	0.018
ESI-	Sphingomyelin	C_47_H_93_N_2_O_6_P	811.669	1.347	8.6	0.039
ESI-	Vanillic acid	C_8_H_8_O_4_	153.019	1.778	0.55	0.006
ESI-	Galactose	C_6_H_12_O_6_	165.04	1.622	0.55	3.20 × 10^−5^
ESI-	Uric acid	C_5_H_4_N_4_O_3_	167.02	1.046	0.55	0.007
ESI-	Yanhusoyl Acetoacetate	C_8_H_8_O_6_	185.01	1.589	0.55	0.003
ESI-	Galactitol	C_6_H_14_O_6_	217.047	2.323	0.55	0.002
ESI-	Inositol phosphate	C_6_H_16_O_18_P_4_	484.901	1.195	0.55	0.026
ESI-	Oxalosuccinic acid	C_6_H_6_O_7_	224.979	1.222	0.56	0.011
ESI-	2′-Deoxyinosine Triphosphate	C_10_H_15_N_4_O_13_P_3_	490.922	1.064	0.56	0.02

In the comparison between the healthy control group and the heat asthma group, 50 differential metabolites were detected: 34 in positive ion mode and 16 in negative ion mode (Figures 2c,d). Compared with the healthy control group, metabolites, including androstenediol, triglyceride, diacylglycerol, and cholesteryl ester, were significantly upregulated in the heat asthma group. The levels of fructose, galactose, uric acid, tetrahydropyridine dicarboxylate, and galactose alcohol were significantly reduced (Table 4).

**Table 4:** List of differentially expressed metabolites in children with heat asthma and healthy controls.

Mode	Metabolites name	Molecular formula	m/z	VIP	Fold change	q-value
ESI+	Fructose	C_12_H_22_O_11_	360.1488	2.375	0.54	0.004
ESI+	2-Hexenyl-6-methoxy-1,4-benzoquinone	C_37_H_54_O_3_	529.4	4.486	0.57	8.15 × 10^−11^
ESI+	Arginine	C_6_H_14_N_4_O_2_	175.1197	1.316	0.67	0.001
ESI+	3-hydroxydecanoyl carnitine	C_18_H_35_NO_5_	328.2461	2.348	0.67	1.06 × 10^−7^
ESI+	Vitamin A	C_20_H_30_O	304.2634	1.903	0.66	0.033
ESI+	Decanoyl carnitine	C_17_H_33_NO_4_	316.2492	2.818	0.68	2.18 × 10^−5^
ESI+	3-hydroxydodecyl carnitine	C_19_H_37_NO_5_	342.2644	2.145	0.72	<0.001
ESI+	Monoacylglycerol ester	C_25_H_38_O_4_	385.2753	1.29	0.72	0.014
ESI+	Corticosterone	C_21_H_34_O_4_	368.2799	2.029	0.75	0.004
ESI+	8-isoprostane	C_20_H_40_	298.347	1.647	1.82	0.013
ESI+	20-hydroxyarachidonic acid	C_20_H_32_O_3_	303.2327	1.697	9.5	0.041
ESI+	Trihexose ceramide	C_52_H_97_NO_18_	1,024.677	1.029	5.82	0.036
ESI+	Androstenediol	C_19_H_30_O	257.2272	1.196	11.3	<0.001
ESI+	Bile acid metabolites	C_27_H_46_O_3_	401.342	1.022	2.98	0.016
ESI+	Valine	C_5_H_11_NO_2_	140.0686	1.254	4.4	0.006
ESI+	Vitamin A2	C_20_H_28_O	285.2229	1.349	9.51	0.021
ESI+	Ganglioside GM3	C_59_H_108_N_2_O_21_	1,181.751	1.028	5.93	0.04
ESI+	Pregnanetriol	C_21_H_36_O_3_	337.2739	1.031	8.38	0.031
ESI+	Ganglioside GA2	C_56_H_104_N_2_O_18_	1,075.728	2.873	6.31	0.024
ESI+	Linolenic acid	C_18_H_30_O_2_	279.2325	1.68	9.55	0.029
ESI+	Leukotriene-B4	C_20_H_32_O_4_	319.2276	1.295	9.55	0.045
ESI+	Lysophosphatidylethanolamine	C_25_H_48_NO_7_P	544.2756	1.257	1.33	0.034
ESI+	Lysophosphatidylcholine	C_22_H_46_NO_7_P	468.3088	1.101	0.96	0.033
ESI+	Cardiolipin	C_73_H_142_O_17_P_2_	1,371.001	1.466	1.5	0.003
ESI+	Triglyceride	C_59_H_96_O_6_	923.717	1.09	10	0.019
ESI+	Diglyceride	C_39_H_66_O_5_	597.4877	3.409	10.1	4.70 × 10^−9^
ESI+	Cholesteryl ester	C_47_H_78_O_3_	729.5615	1.733	9.96	0.012
ESI+	Ceramide	C_44_H_87_NO_3_	716.633	1.249	10.92	0.022
ESI+	Phosphatidylglycerol	C_46_H_81_O_10_P	842.5897	2.77	3.21	0.033
ESI+	Phosphatidylethanolamine	C_39_H_78_NO_7_P	686.5544	1.308	9.93	0.024
ESI+	Monoacylglycerol ester	C_21_H_38_O_4_	355.286	3.564	6.3	5.68 × 10^−6^
ESI+	Galactosylceramide	C_40_H_77_NO_8_	682.5609	1.204	6.66	0.029
ESI+	Sphingomyelin	C_45_H_89_N_2_O_6_P	807.6351	1.126	7.32	0.03
ESI+	Phosphatidylinositol	C_47_H_87_O_13_P	908.6153	1.218	8.54	0.019
ESI-	Phosphatidylethanolamine	C_45_H_84_NO_7_P	780.5893	1.139	8.01	0.031
ESI-	Phosphatidylinositol	C_49_H_83_O_13_P	909.551	1.36	5.58	0.02
ESI-	Phosphatidylcholine	C_46_H_86_NO_7_P	780.5893	1.139	8.01	0.031
ESI-	Vanillic acid	C_8_H_8_O_4_	153.0187	2.023	0.55	0.019
ESI-	Galactose	C_6_H_12_O_6_	165.0398	1.671	0.55	0.007
ESI-	Uric acid	C_5_H_4_N_4_O_3_	167.0201	1.218	0.55	0.046
ESI-	Tetrahydropyridine dicarboxylate	C_7_H_9_NO_4_	170.045	1.407	0.55	0.003
ESI-	Galactitol	C_6_H_14_O_6_	217.0468	1.266	0.55	0.023
ESI-	Taurine	C_2_H_7_NO_3_S	124.0079	1.15	0.56	0.019
ESI-	Biotin	C_10_H_16_N_2_O_3_S	279.0597	2.077	0.56	0.034
ESI-	Dihydropterin triphosphate	C_9_H_16_N_5_O_13_P_3_	479.9686	2.069	0.58	0.039
ESI-	Leukotriene-B4	C_20_H_32_O_4_	335.222	2.895	0.76	0.035
ESI-	2-Stearylglycerol phosphate inositol	C_27_H_53_O_12_P	599.3194	1.885	1.32	0.009
ESI-	Glucosylceramide	C_46_H_89_NO_8_	782.6504	1.278	8.63	0.029
ESI-	Lysophosphatidylethanolamine	C_23_H_46_NO_7_P	478.2927	2.045	0.65	0.026
ESI-	Triglyceride	C_59_H_94_O_6_	883.6883	1.332	8.53	0.01

Due to the limited number of differential metabolites identified between the cold and heat asthma groups in the negative ion mode (Supplementary Figures S1D,F), further analysis was conducted exclusively in the positive ion mode (Figure 2e). A total of 18 differential metabolites were identified. Among them, vitamin A, androstenol, and triglycerides were significantly upregulated in the heat asthma group compared with in those the cold asthma group (Table 5).

**Table 5:** List of differentially expressed metabolites in children with cold asthma and heat asthma syndrome.

No.	Metabolites name	Molecular formula	m/z	VIP	Fold change	q-value
1	8-isoprostane	C_20_H_40_	298.347	2.033	1.82	0.039
2	Vitamin A	C_20_H_30_O	287.236	1.379	11.31	0.025
3	Ganglioside GM3	C_64_H_120_N_2_O_21_	1,235.843	1.085	7.23	0.034
4	Trihexose ceramide	C_56_H_105_NO_18_	1,102.721	2.569	9.13	0.019
5	Valine	C_5_H_11_NO_2_	140.069	2.207	4.41	0.013
6	Taurine	C_18_H_32_O_2_	263.238	1.22	6.16	0.018
7	4,4-Dimethyl-5α-cholest-8-en-3B-ol	C_29_H_50_O	397.383	1.251	11.27	0.035
8	Androstenol	C_19_H_30_O	257.227	1.391	11.31	0.018
9	Retinoic acid coenzyme A	C_18_H_32_O_2_	1,072.306	1.22	8.17	0.018
10	α-Tocopherol-succinate	C_33_H_54_O_5_	548.429	1.168	8.6	0.018
11	Triglycerides	C_68_H_120_O_6_	1,055.899	1.053	10.96	0.025
12	Leukotriene	C_20_H_32_O_4_	319.228	1.541	8.8	0.013
13	Diacylglycerol	C_42_H_70_O_5_	637.515	1.359	6.53	0.04
14	Phosphatidylethanolamine	C_34_H_68_NO_8_P	672.456	2.043	6.12	0.011
15	Phosphatidylcholine	C_48_H_80_NO_8_P	847.595	1.787	5.51	0.039
16	Phosphatidylinositol	C_45_H_81_O_13_P	899.602	1.491	7.24	0.006
17	7-α, 27-dihydroxy-cholesterol	C_27_H_46_O_3_	419.352	1.327	8.58	0.018
18	Octadecanoic acid	C_18_H_34_O_3_	299.259	1.419	8.48	0.027

### 3.4 Identification of differentially enriched metabolic pathways

**Figure 3:**
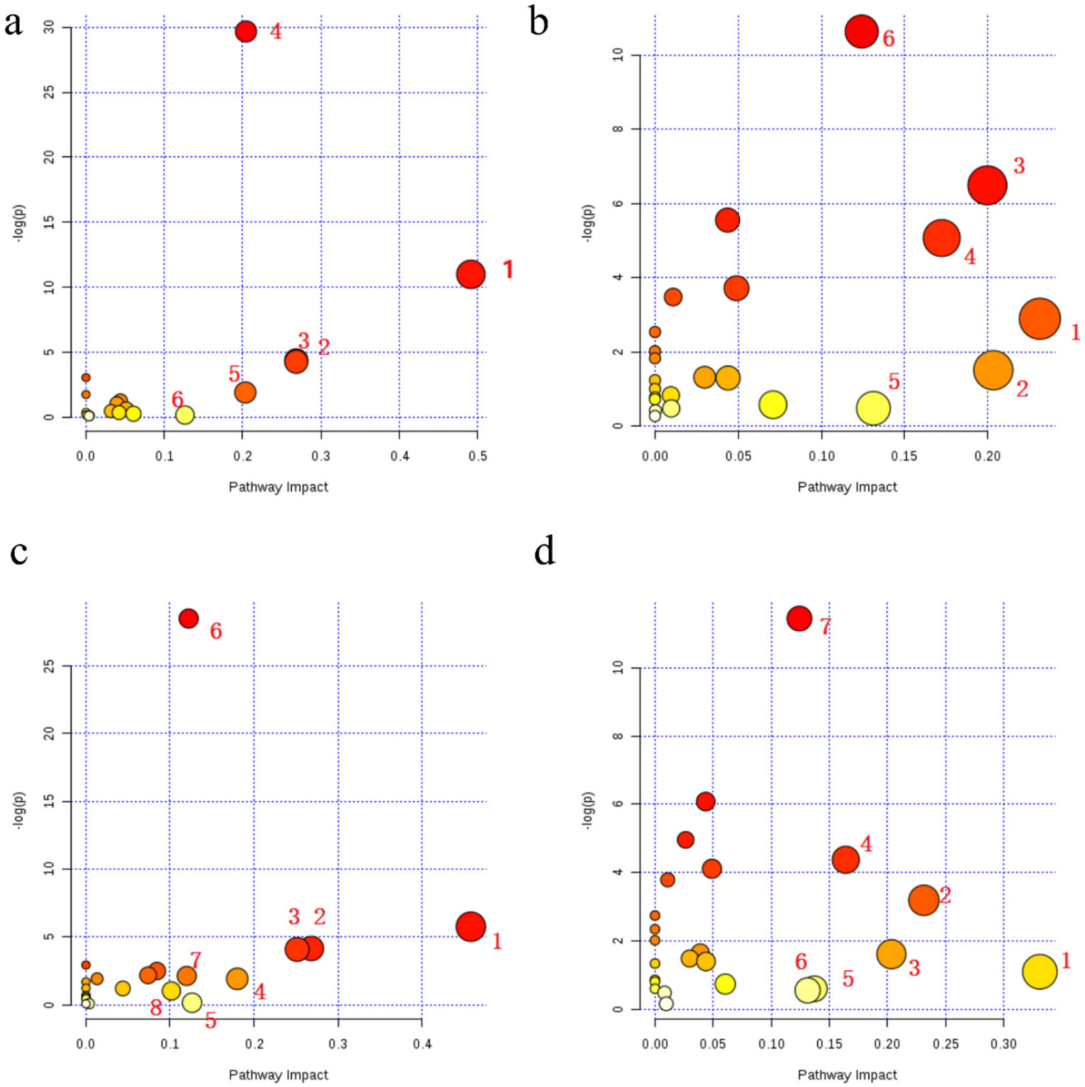
Bubble charts of differential metabolic pathways between asthma subtypes and healthy controls in both ionization modes. **(a,b)** Differential metabolic pathways between the healthy control and the cold asthma groups in positive ion mode **(a)** and negative ion mode **(b)**. **(c,d)** Differential metabolic pathways between the healthy control group and the heat asthma group in the positive ion mode **(c)** and negative ion mode **(d)**.

To identify differentially enriched metabolic pathways, MetaboAnalyst 4.0 platform (https://www.metaboanalyst.ca/) was used. In the positive ion mode, six metabolic pathways were found to be significantly altered in the healthy control and cold asthma groups. These included the sphingolipid, retinol, glycerophospholipid, arachidonic acid, linolenic acid, arginine, and proline metabolism pathways (Figure 3a, Table 6). Six additional differential pathways were identified in negative ion mode: glycerophospholipid metabolism, biotin metabolism, phosphoinositide metabolism, tyrosine metabolism, folate biosynthesis, and galactose metabolism (Figure 3b, Table 6).

**Table 6:** Differential metabolic pathways between children with cold asthma and the healthy controls in both the positive and negative ion modes.

Ion modes	No.	Pathway name	*p* value	−log(*p*)	FDR	Impact
Positive	1	Sphingolipid metabolic pathway	1.69 × 10^−5^	10.9	6.76 × 10^−4^	0.49
	2	Retinol metabolism	0.014	4.26	0.282	0.27
	3	Glycerophospholipid metabolism	0.013	4.38	0.282	0.27
	4	Arachidonic acid metabolism	1.29 × 10^−13^	29.7	1.03 × 10^−11^	2.00 × 10^−1^
	5	Linolenic acid metabolism	0.149	1.9	1	0.2
	6	Arginine and proline metabolism	0.847	0.17	1	0.13
Negative	1	Glycerophospholipid metabolism	0.055	2.9	0.632	0.23
	2	Biotin metabolism	0.221	1.51	1	0.2
	3	Phosphoinositide metabolism	0.002	6.49	0.061	0.2
	4	Tyrosine metabolism	0.006	5.07	0.126	0.17
	5	Folic acid biosynthesis	0.618	0.48	1	0.13
	6	Galactose metabolism	2.40 × 10^−5^	10.64	0.002	0.12

Eight differential metabolic pathways were identified in the positive ion mode between the healthy control and heat asthmatic groups. These included sphingolipids, retinol, glycerophospholipid, pantothenate and CoA biosynthesis, arginine and proline, arachidonic acid, glycine/serine/threonine, and starch and sucrose metabolism (Figure 3c, Table 6). Seven altered pathways were observed in negative ion mode: taurine metabolism, glycerophospholipid metabolism, biotin metabolism, sphingolipid metabolism, phosphoinositide metabolism, folate biosynthesis, and galactose metabolism (Figure 3d, Table 7).

**Table 7:** Differential metabolic pathways between children with heat asthma and healthy controls in both positive and negative ion modes.

Ion modes	No.	Pathway name	*p* value	−log(*p*)	FDR	Impact
Positive	1	Sphingolipid metabolism	0.003	5.75	0.127	0.46
	2	Glycerophospholipid metabolism	0.016	4.15	0.339	0.27
	3	Retinol metabolism	0.017	4.08	0.339	0.25
	4	Pantothenate and COA biosynthesis	0.148	1.91	1	0.18
	5	Arginine and proline metabolism	0.866	0.14	1	0.13
	6	Arachidonic acid metabolism	4.20 × 10^−13^	28.5	3.36 × 10^−11^	1.20 × 10^−1^
	7	Glycine, serine, threonine metabolism	0.12	2.12	1	0.12
	8	Starch and sucrose metabolism	0.364	1.01	1	0.1
Negative	1	Taurine metabolism	0.333	1.1	1	0.33
	2	Glycerophospholipid metabolism	0.041	3.19	0.471	0.23
	3	Biotin metabolism	0.199	1.61	1	0.2
	4	Sphingolipid metabolism	0.013	4.38	0.252	0.16
	5	Phosphoinositide metabolism	0.547	0.6	1	0.14
	6	Folic acid biosynthesis	0.574	0.56	1	0.13
	7	Galactose metabolism	1.08 × 10^−5^	11.44	8.61 × 10^−4^	1.20 × 10^−1^

**Figure 4:**
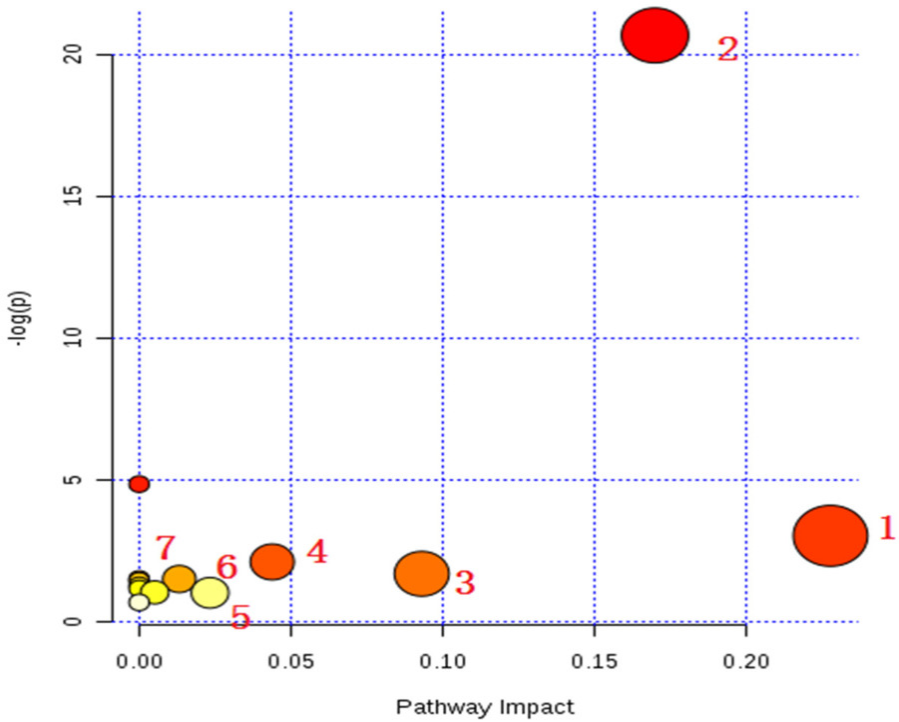
Bubble chart of differential metabolic pathways between cold asthma and heat asthma in the positive ion mode.

Further analysis of the differential pathways between the cold asthma and heat asthma groups revealed seven significantly enriched metabolic pathways: glycerophospholipid metabolism, arachidonic acid metabolism, retinol metabolism, glycosylation biosynthesis, glycine/serine/threonine metabolism, valine/leucine/isoleucine metabolism, and the protobiliary acid biosynthesis pathway (Figure 4, Table 8).

**Table 8:** Differential metabolic pathways between children with cold asthma and heat asthma syndrome in the positive ion mode.

No.	Pathway name	*p* value	−log(*p*)	FDR	Impact
1	Glycerophospholipid metabolic pathway	0.048	3.03	1	0.23
2	Arachidonic acid metabolic pathway	1.06 × 10^−9^	20.7	8.44 × 10^−8^	1.70 × 10^−1^
3	Retinol metabolism	0.184	1.7	1	0.09
4	Glycosylation biosynthesis pathway	0.121	2.11	1	0.04
5	Glycine, serine, threonine metabolism	0.359	1.04	1	0.02
6	Metabolism of valine, leucine and isoleucine	0.221	1.51	1	0.01
7	Protobiliary acid biosynthesis pathway	0.353	1.04	1	0.01

In summary, sphingolipid metabolism, retinol metabolism, glycerophospholipid metabolism, and arachidonic acid metabolism pathways distinguished both cold and heat asthma groups from healthy controls. Meanwhile, metabolites in glycerophospholipid metabolism and glycosylation biosynthesis pathways may serve as distinguishing biomarkers between cold and heat asthma syndromes.

## 4 Discussion

This study presents a comprehensive untargeted metabolomic analysis of plasma samples from children with cold asthma syndrome, heat asthma syndrome, and healthy controls. Several metabolites were differentially expressed between the groups. These findings may help to elucidate the underlying mechanisms of cold and heat asthma syndromes and support the identification of potential biomarkers for TCM syndrome differentiation.

Fifty metabolites were identified as significantly altered in patients with cold asthma syndrome compared with healthy controls. These changes included changes in fructose, lipids and their derivatives, amino acids, phospholipids, and related metabolites. Notably, the levels of trihexosylceramide, androstenediol, triglyceride, diglyceride, and isodeoxycholic acid were elevated by more than 10-fold in the cold asthma group relative to controls. Additionally, levels of monoacylglycerides, prostaglandin A1, 20-hydroxyarachidonic acid, pregnanetriol, vitamin A2, cholesteryl ester, phosphatidylethanolamine (PE), and sphingomyelin (SM) were increased by more than 9-fold in children with cold asthma syndrome compared to healthy children.

Kyoto Encyclopedia of Genes and Genomes (KEGG) pathway analysis revealed that these differential metabolites were primarily enriched in sphingolipid, glycerophospholipid, arachidonic acid (AA), arginine and proline, and tyrosine metabolism. Previous studies have shown that lipid metabolism is closely associated with airway hyper-responsiveness and an increased risk of asthma and wheezing attacks (25). Free fatty acid receptor 1 (GPR40), which is expressed on airway smooth muscle cells, functions as a sensor for medium- and long-chain free fatty acids. Its activation can promote airway smooth muscle cell proliferation and p70S6 K phosphorylation via the MEK/ERK and PI3 K/Akt signaling pathways, thereby aggravating asthma severity (26).

In the present study, we also observed significant changes in plasma lipid metabolites in children with both cold and heat asthma syndromes. Consistent with our results, Jiang et al. (16) reported disturbed lipid metabolism in asthmatic patients, characterized by significantly higher levels of triglycerides, ceramides (Cer), PE, SM, phosphatidylinositol (PI), phosphatidylglycerol (PG), and lysophosphatidylcholine (LPC), than in healthy controls. Similarly, Wang et al. (17, 18) found significant alterations in plasma levels of phosphatidic acid (PA), PG, PE, phosphatidylcholine (PC), PI, and phosphatidylserine in patients with asthma. Furthermore, several metabolomic studies have demonstrated that the incidence and severity of asthma in children are closely associated with abnormalities in fatty acid, glycerophospholipid, and sphingolipid metabolism (27). Our findings are consistent with those of previous research and further support the presence of metabolic dysregulation in pediatric asthma.

However, to date, no study has directly linked lipid metabolism with TCM syndromes to provide mechanistic insights supporting the TCM theory. According to TCM, typical clinical manifestations of cold asthma syndrome include wheezing in the throat, shortness of breath, chest tightness, white frothy sputum, absence of thirst or a preference for warm drinks, cold limbs, bluish facial complexion, and a tendency for symptom exacerbation upon exposure to cold environments. These manifestations may reflect the underlying metabolic disturbances in the affected children. As asthma is primarily a respiratory disease, most symptoms are concentrated in the airway. However, children with cold asthma syndrome often present with cold intolerance, peripheral coldness, and a dull or cyanotic complex, suggesting reduced metabolic activity and impaired systemic homeostasis. Exposure to cold or other environmental triggers can cause acute exacerbations. Our current study found that children with cold asthma syndrome exhibited significant metabolic alterations, with markedly increased plasma levels of metabolites, such as androstenediol, triglycerides, diglycerides, isodeoxycholic acid, monoacylglycerides, and prostaglandin A1.

Similarly, in the heat asthma syndrome group, plasma levels of androstenediol, triglycerides, diglycerides, and ceramide were elevated more than 10-fold compared to controls. Moreover, 20-hydroxyarachidonic acid, vitamin A2, linolenic acid, leukotriene B4, cholesteryl esters, and PE increased by more than 9-fold. KEGG pathway enrichment analysis indicated that these differential metabolites were associated with sphingolipid, glycerophospholipid, taurine, arginine and proline, glycine, serine, and threonine metabolism.

Adipose tissue functions as a metabolically active endocrine organ and plays a central role in the regulation of energy balance and metabolic homeostasis. The hydrolysis of triglycerides and the release of free fatty acids (FFAs) from adipocytes, especially when insulin-mediated, can activate the MAPK and PI3 K signaling pathways. This promotes smooth muscle proliferation in the airway, exacerbating asthma symptoms (26).

In TCM, heat asthma syndrome is characterized by symptoms such as loud phlegm sounds in the throat, shortness of breath, persistent coughing with thick yellow or white sputum, a dry or bitter taste in the mouth, facial flushing, sweating, and body heat. Our findings suggest that children with heat asthma also exhibit marked metabolic imbalance. The differentially expressed metabolites in this group included androstenediol, triglycerides, diglycerides, ceramide, 20-hydroxyarachidonic acid, vitamin A2, linolenic acid, and leukotriene B4, all of which may contribute to the pathophysiological basis of the heat syndrome as interpreted through TCM principles.

A comparative analysis between children with cold asthma and those with heat asthma syndromes revealed that several metabolites were significantly elevated in the cold asthma group. Specifically, the levels of vitamin A, 4,4-dimethyl-5α-cholyl-8-ene-3β-alcohol, androstenediol, and triglycerides were more than 10-fold higher in patients with cold asthma syndrome than in those with heat asthma syndrome. Additionally, trihexosylceramide, retinoic acid coenzyme A, α-tocopherol succinate, leukotriene, 7-α,27-dihydroxycholesterol, and octadecanoic acid were elevated by more than 8-fold in the cold asthma group.

Pathway enrichment analysis indicated that these differential metabolites are primarily involved in glycerophospholipid metabolism, arachidonic acid, retinol, glycosylation, glycine/serine/threonine, valine/leucine/isoleucine, and primary bile acid biosynthesis. Wang et al. (28) reported that cysteine and glycine-rich protein 2 (Csrp2) plays a key role in the phenotypic switching of airway smooth muscle cells (ASMCs). Upregulation of Csrp2 inhibits the PDGF-BB-induced transition of ASMCs to a synthetic/proliferative phenotype via modulation of the Yes-associated protein (YAP)/transcriptional coactivator with PDZ-binding motif (TAZ) signaling axis, thereby contributing to airway remodeling in asthma. Kelly et al. (29) conducted a systematic review of 21 studies utilizing metabolomics techniques and concluded that asthma pathogenesis is closely associated with various metabolic pathways, including amino acid metabolism, glutamate-glutamine cycle, hypoxia response, immune and inflammatory signaling, lipid metabolism, oxidative stress, and tricarboxylic acid (TCA) cycle. These findings support the notion that correcting metabolic imbalance may be a promising therapeutic approach for asthma. Furthermore, Gao et al. (30) used ultra-high-performance liquid chromatography–mass spectrometry to analyze serum samples from children with asthma and bronchiolitis. Their results showed significant alterations in the serum levels of phosphatidylethanolamine, saturated and monounsaturated fatty acids, and bile acids in asthmatic children.

In our comparative analysis of cold and heat asthma syndromes, we identified several common differentially expressed serum metabolites, including triglycerides, phosphatidylethanolamine, and ceramides. These shared biomarkers suggest that despite differences in syndrome classification, both cold and heat asthma share core respiratory pathophysiological features. However, distinct metabolic profiles between the two syndromes likely reflect underlying differences in etiology and clinical presentation, as described in TCM theory.

Compared with Western medicine, Traditional Chinese Medicine (TCM) has been widely applied in the treatment of asthma, chronic obstructive pulmonary disease (COPD), and other diseases owing to its holistic and systemic approach. Clinical practice has demonstrated that TCM offers notable advantages, including significant efficacy and favorable safety profile. TCM provides unique therapeutic benefits in asthma. According to TCM theory, asthma falls under the category of “*Xiao*” (喘证). However, the classification of bronchial asthma into different syndrome types in TCM is often subjective and heavily reliant on individual physician experience and lacks an objective and standardized basis for syndrome differentiation. This subjectivity has hindered the establishment of standardized diagnostic and treatment protocols. Moreover, relatively few studies have systematically explored the theoretical underpinnings and biological validation of TCM syndromes, particularly those related to asthma.

In our study, we observed significant differences in sphingolipid metabolites, including ganglioside GA2, ganglioside GM3, galactosylceramide, and sphingomyelin, between patients with asthma and healthy controls. Sphingolipids are complex lipids comprising a sphingosine backbone, long-chain fatty acids, and polar head groups. They are classified as sphingomyelins, gangliosides, and glycosphingolipids. Alterations in sphingolipids, such as ceramides, have been shown to influence the biosynthesis of long-chain fatty acids, including palmitic acid, which is associated with asthma severity (31). Notably, sphingosine-1-phosphate and ceramide, key metabolites derived from sphingomyelin, have been shown to interact directly with bronchial smooth muscle cells, promoting contraction and contributing to airway remodeling (32, 33).

In addition, our findings demonstrated that the levels of several glycerophospholipids, including lysophosphatidylethanolamine, lysophosphatidylcholine, cardiolipin, PE, PI, and PC, were significantly altered in the plasma of asthma syndrome groups compared with controls. Glycerophospholipids are essential components of cellular membranes and are involved in the synthesis of pulmonary surfactants. Approximately 85% of phospholipids in alveolar surfactants consist of phosphatidylcholine, which plays a critical role in pulmonary defense and is implicated in chronic airway inflammation and increased airway responsiveness (34).

Asai et al. (35) used LC-MS-based metabolomics to analyze bronchoalveolar lavage fluid from asthma patients and found elevated levels of lysophosphatidylcholine and phospholipase A2 activity compared to healthy controls. Lysophosphatidylcholine exerts strong pro-inflammatory effects and contributes to alveolar epithelial cell injury. The latest research indicated that lysophosphatidylcholine may serve as novel lipid mediators of severe asthma involving to corticosteroid resistance and correlating with oxidative stress (36), of which once again proves the importance of the current research findings.

Interestingly, in our comparative analysis of the cold and heat asthma syndrome groups, we also found differential expressions of PE, PC, and PI, suggesting that alterations in the glycerophospholipid metabolism pathway may serve as potential biomarkers for distinguishing between cold and heat asthma syndromes.

Significant alterations in amino acid metabolic pathways were observed between the cold and heat asthma groups. Arginine, a semi-essential amino acid, plays a key role in asthma pathophysiology. Studies have shown that excessive arginine methylation can trigger asthma attacks (37), and the levels of asymmetric dimethylarginine and methylarginine are significantly elevated in patients with severe asthma (38). Arginine is metabolized by arginase to produce endogenous nitric oxide (NO), which modulates airway responsiveness (39). Several studies have demonstrated a correlation between endogenous NO levels and airway hyper-reactivity (40, 41). Conversely, arginase activity has been shown to mitigate airway hyper-responsiveness by reducing systemic arginine levels (42).

Proline, a non-essential cyclic amino acid, also contributes to asthma pathogenesis. The collagen-derived peptide proline-glycine-proline has been shown to induce neutrophil chemotaxis, a key feature of airway inflammation (43). Glycine reduces lung injury by attenuating the recruitment of inflammatory cells and suppressing the release of inflammatory mediators (44). Serine, which participates in the biosynthesis of purines, pyrimidines, glycerophospholipids, and sphingolipids, is involved in the oxidative stress pathways linked to asthma (45). Furthermore, plasma levels of branched-chain amino acids (BCAAs), such as valine, leucine, and isoleucine, were found to be significantly elevated in patients with asthma compared to healthy controls (46). Taken together, the differences in plasma metabolites between children with cold and heat asthma syndromes are not limited to lipids, such as triglycerides, diglycerides, and ceramides, but also extend to amino acids, including arginine, proline, and valine, and their related metabolic pathways.

Overall, our study suggests that serum metabolite profiling in children with cold- and heat-asthma syndromes has the potential to identify biomarkers that support the diagnosis and differentiation of TCM syndromes. These findings could assist TCM practitioners in achieving more accurate syndrome classification for childhood asthma. Our results demonstrate that systemic metabolic disturbances, particularly in lipid and amino acid pathways, are evident in pediatric asthma and that these disturbances differ between cold and heat syndromes. Table 9 summarized the common differential metabolites between cold asthma and heat asthma, cold asthma and the healthy group, and heat asthma and the healthy group. These common differential metabolites were mainly lipids, amino acids and its related metabolite such as triglyceride, diglyceride and arginine, ceramide that can be considered as biomarkers. In addition, through comparative analysis, we also concluded in Table 10 that the other potential plasma differential metabolites biomarkers and the metabolic pathways can be used in differential diagnosis of cold and heat asthma syndromes.

**Table 9:** Potential biomarkers in diagnosis of TCM cold and heat asthma syndrome.

m/z	Metabolites	Molecular formula	*m*/*z*	Metabolites	Molecular formula
360.149	Fructose	C_12_H_22_O_11_	923.717	Triglyceride	C_59_H_96_O_6_
529.4	2-Hexenyl-6-methoxy-1,4-benzoquinone	C_37_H_54_O_3_	597.488	Diglyceride	C_39_H_66_O_5_
175.12	Arginine	C_6_H_14_N_4_O_2_	729.561	Cholesteryl ester	C_47_H_78_O_3_
328.246	3-hydroxydecanoyl carnitine	C_18_H_35_NO_5_	872.653	Ceramide	C_48_H_91_NO_13_
304.263	Vitamin A	C_20_H_30_O	842.59	Phosphatidylglycerol	C_46_H_81_O_10_P
316.249	Decanoyl carnitine	C_17_H_33_NO_4_	750.541	Phosphatidylethanolamine	C_43_H_78_NO_8_P
342.264	3-hydroxydodecyl carnitine	C_19_H_37_NO_5_	279.06	Biotin	C_10_H_16_N_2_O_3_S
337.273	Monoacylglycerol ester	C_21_H_38_O_4_	599.319	2-Stearylglycerol phosphate inositol	C_27_H_53_O_12_P
1,075.728	Ganglioside GA2	C_56_H_104_N_2_O_18_	478.293	Lysophosphatidylethanolamine	C_23_H_46_NO_7_P
368.28	Corticosterone	C_21_H_34_O_4_	948.722	Phosphatidylethanolamine	C_53_H_104_NO_8_P
303.233	20-hydroxyarachidonic acid	C_20_H_32_O_3_	744.556	Phosphatidylcholine	C_41_H_80_NO_8_P
1,097.726	Trihexose ceramide	C_54_H_99_NO_18_	811.669	Sphingomyelin	C_47_H_93_N_2_O_6_P
285.223	Vitamin A2	C_20_H_28_O	355.286	Monoacylglycerol ester	C_21_H_38_O_4_
337.273	Pregnanetriol	C_21_H_36_O_3_	682.561	Galactosylceramide	C_40_H_77_NO_8_
301.212	Linolenic acid	C_18_H_30_O_2_	153.019	Vanillic acid	C_8_H_8_O_4_
544.278	Lysophosphatidylethanolamine	C_25_H_48_NO_7_P	165.04	Galactose	C_6_H_12_O_6_
468.309	Lysophosphatidylcholine	C_22_H_46_NO_7_P	167.02	Uric acid	C_5_H_4_N_4_O_3_
1,371.001	Cardiolipin	C_73_H_142_O_17_P_2_	217.047	Galactitol	C_6_H_14_O_6_

**Table 10:** The other potential plasma differential metabolites biomarkers and related metabolic pathways between cold and heart asthma.

TCM syndrome	Metabolites name	Molecular formula	Metabolic pathways
Cold asthma	2′-deoxycytidine 5′-triphosphate	C_9_H_16_N_3_O_13_P_3_	Linolenic acid metabolism Tyrosine metabolism Glycosylation biosynthesis pathway Metabolism of valine, leucine and isoleucine Protobiliary acid biosynthesis pathway
	Prostaglandin A1	C_20_H_32_O_4_	
	Androstenol	C_19_H_30_O	
	Pregnanetriol	C_21_H_36_O_3_	
	3-Hydroxy-9-Octadecyl Carnitine	C_25_H_47_NO_5_	
	Lactoside ceramide	C_48_H_91_NO_13_	
	Biotin	C_10_H_16_N_2_O_3_S	
	Isodeoxycholic acid	C_24_H_40_O_4_	
	Ganglioside GA2	C_56_H_104_N_2_O_18_	
	Phosphatidylserine	C_42_H_82_NO_10_P	
	Uric acid	C_5_H_4_N_4_O_3_	
	Yanhusoyl Acetoacetate	C_8_H_8_O_6_	
	Inositol phosphate	C_6_H_16_O_18_P_4_	
	Oxalosuccinic acid	C_6_H_6_O_7_	
	2 ‘- Deoxyinosine Triphosphate	C_10_H_15_N_4_O_13_P_3_	
Heat asthma	8-isoprostane	C_20_H_40_	Pantothenate and COA biosynthesis Glycine, serine, threonine metabolis Starch and sucrose metabolism Taurine metabolism
	Phosphatidylinositol	C_47_H_87_O_13_P	
	Phosphatidylinositol	C_49_H_83_O_13_P	
	Taurine	C_2_H_7_NO_3_S	
	Leukotriene-B4	C_20_H_32_O_4_	
	Glucosylceramide	C_46_H_89_NO_8_	

In addition, attentions should also be paid to the dramatically change of arachidonic acid (AA) metabolic pathway and retinol metabolism. Asthma, as a chronic disease, is characteristic with airway inflammation, airway hyperresponsiveness, mucus overproduction, remodeling, and narrowing of the airway. Studies have demonstrated that inflammatory characteristics of asthma may be related to the release of Th2 cytokines (IL-4, IL-5, IL-13 and IL-33) mediated by the regulation of arachidonic acid metabolic pathway (17, 18, 47). Meanwhile, the cyclooxygenase (COX) and lipoxygenase (LOX) pathways involved in AA metabolism are also involved in lipid synthesis and metabolism (17, 18, 48). These evidences indicated that AA metabolism may link to the lipid synthesis and metabolism during the asthma onset process, but there still lack of evidence used to explain its relationship with the classification of TCM asthma syndromes. Another important issue is retinol metabolism. Retinol, also named vitamin A, was change significantly in both syndromes despite distinct clinical presentations in the current study. As is well known, vitamins including fat-soluble and water-soluble kinds, a type of trace organic substances, are indispensable to human healthy and primarily obtained from food, mainly participate in biochemical reactions and regulate metabolic functions (49, 50). In our study, vitamin A and vitamin A2 were identified as differential metabolites, and retinol metabolism is one of the differential metabolic pathways. Dominika et al. systematically summarized that the role of vitamins in the pathogenesis of asthma including the influence of vitamins on asthma and its typical symptoms (51). They also summarized the correlation between vitamin intake and levels and the risk of asthma in both pre- and postnatal life. Hu and Sang reported that no significant correlation between vitamin A intake and asthma risk in children, nor between serum vitamin A levels and asthma risk (52,53). Taken together, the evidence regarding vitamins and asthma is still controversial due to the complex pathogenesis of asthma. In other words, the intake of vitamin and its balances can be considered as an intervention to prevent asthma indicated by our study and others.

However, this study has several limitations. First, the sample size was relatively small, which may have limited the generalizability of the findings and may bring bias to the research results. Second, the absence of a drug intervention group prevented us from evaluating the therapeutic effect of TCM treatment and its influence on the identified metabolic markers. In the future studies, we not only should expand the sample size, but also increase the study before and after the intervention of TCM formulas (such as classic formulas). At the same time, we should use high-throughput technologies, and combine leverage big data with artificial intelligence technology to construct a plasma biomarker model for asthma patients with cold or heat asthma syndrome based on the key findings, and conduct large-scale and multi- center clinical data validation studies. Moreover, mature *in vivo* animal models and *in vitro* cell experiments are also necessary performed to elucidate in-depth on its mechanisms, providing comprehensive theoretical basis for the clinical treatment of asthma patients with different TCM syndromes. Finally, the comparison between cold and heat asthma syndromes was primarily based on positive-ion mode data. Limited information is available from the negative ion mode, which may have omitted additional relevant metabolic alterations.

## 5 Conclusion

Our study provides important insights into the plasma differential metabolites associated with TCM-defined asthma syndromes in children, particularly distinguishing between cold and heat syndromes. These findings contribute to the modern scientific understanding of TCM syndrome classification in asthma. The common lipids, amino acids, and their associated metabolic pathways have emerged as potential biomarkers for the differential diagnosis of cold and heat syndromes in TCM. Key lipid-related metabolites and pathways include triglycerides, diglycerides, sphingomyelin, phosphatidylcholine, sphingolipid metabolism, glycerophospholipid metabolism, and phosphoinositide metabolism. Significant contributors of amino acids include taurine, valine, and arginine, along with the glycine/serine/threonine metabolism, taurine metabolism, and arginine/proline metabolism pathways. These results enhance our understanding of metabolic dysregulation in children with cold or heat asthma syndromes and offer a scientific basis for exploring its pathogenesis, improving clinical syndrome differentiation, and evaluating the therapeutic efficacy of TCM interventions. Future studies should further explore amino acid metabolism in children with different TCM asthma syndromes and identify characteristic amino acid markers that reflect TCM syndrome differentiation with greater objectivity.

## Data Availability

The raw data supporting the conclusions of this article will be made available by the authors, without undue reservation.
