# Supplementary material for: Plasma lipids, amino acids, and their metabolic pathways as potential biomarkers for differential diagnosis of cold and heat syndrome asthma in children: a preliminary study
Source: Front Pediatr. 2025 Jul 23;13:1549431. doi: 10.3389/fped.2025.1549431 (PMC12325226; doi:10.3389/fped.2025.1549431)
Supplement: Supplementary file 1 [file Supplementaryfile1.docx]

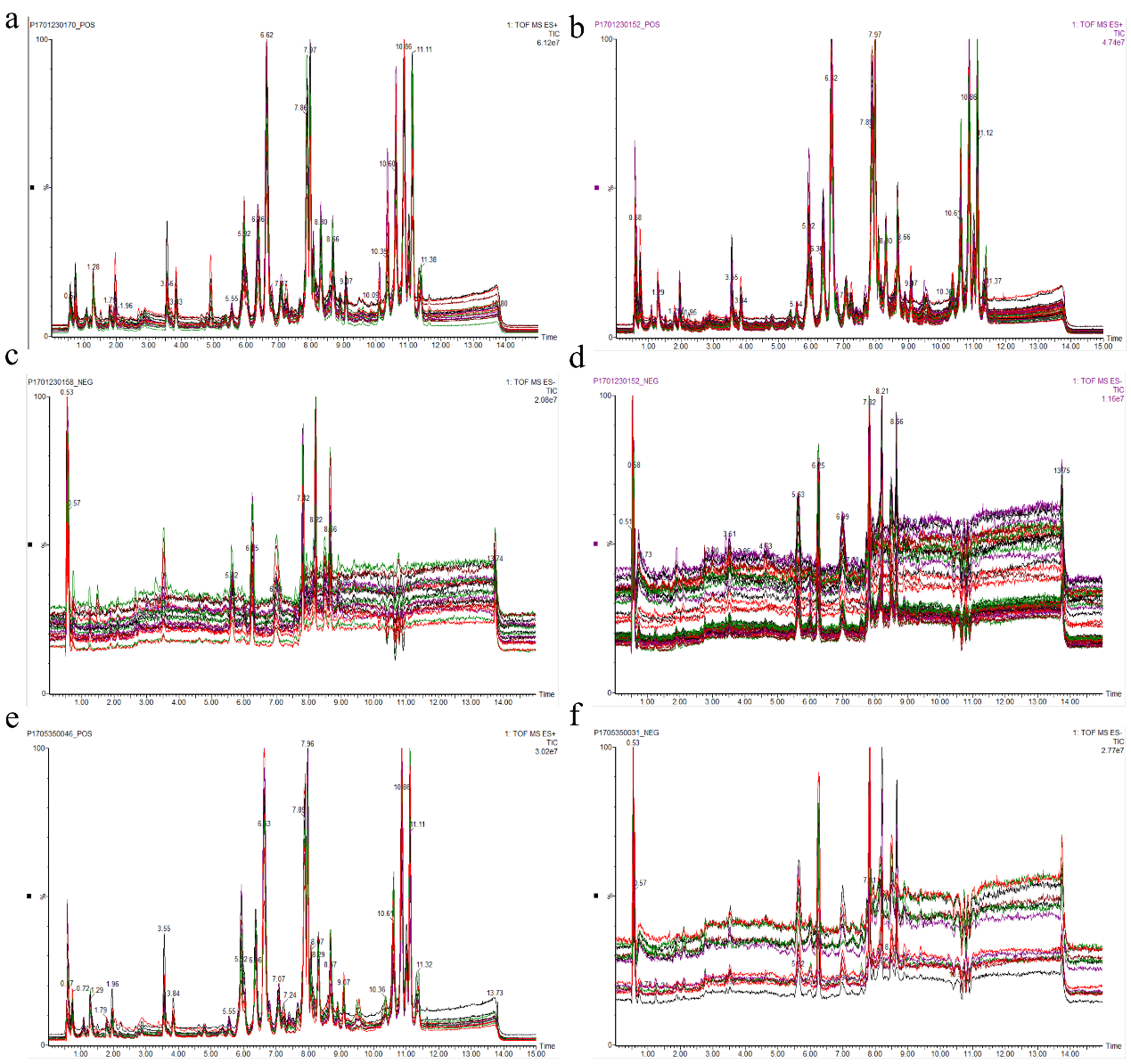


**Supplementary Figure 1.** Total ion chromatogram (TIC) of the healthy controls, cold asthma, heat asthma and quality control (QC) samples under positive and negative ions models. (a) TIC results between quality control and healthy controls in positive ion model. (b) TIC results between quality control and cold asthma in positive ion model. (c) TIC results between quality control and healthy controls in negative ion model. (d) TIC results between quality control and cold asthma in negative ion model. (e) TIC results between quality control and heat asthma in positive ion model. (f) TIC results between quality control and heat asthma in negative ion model. In the TIC plots: the abscissa represents the retention time, and the ordinate represents the ionic strength, which reflects the change rule of each component in the plasma sample with the retention time, and represents the metabolite spectrum peaks detected at different retention times and their ionic strength.
